# Repeated occurrence of severe hypotension associated with azithromycin infusion in a patient with fulminant myocarditis: a case report

**DOI:** 10.1093/ehjcr/ytae208

**Published:** 2024-04-18

**Authors:** Max Lenz, Konstantin A Krychtiuk, Robert Zilberszac, Walter S Speidl, Gottfried Heinz

**Affiliations:** Division of Cardiology, Department of Internal Medicine II, Medical University of Vienna, Waehringer Guertel 18-20, 1090 Vienna, Austria; Ludwig Boltzmann Institute for Cardiovascular Research, Waehringer Guertel 18-20, 1090 Vienna, Austria; Division of Cardiology, Department of Internal Medicine II, Medical University of Vienna, Waehringer Guertel 18-20, 1090 Vienna, Austria; Ludwig Boltzmann Institute for Cardiovascular Research, Waehringer Guertel 18-20, 1090 Vienna, Austria; Division of Cardiology, Department of Internal Medicine II, Medical University of Vienna, Waehringer Guertel 18-20, 1090 Vienna, Austria; Division of Cardiology, Department of Internal Medicine II, Medical University of Vienna, Waehringer Guertel 18-20, 1090 Vienna, Austria; Division of Cardiology, Department of Internal Medicine II, Medical University of Vienna, Waehringer Guertel 18-20, 1090 Vienna, Austria

**Keywords:** Azithromycin, Fulminant myocarditis, Cardiogenic shock, ICU, ECMO, Case report

## Abstract

**Background:**

Intravenous administration of azithromycin has been linked to severe hypotension in some case reports in the past. We report a further case of profound shock requiring excessive use of vasopressors and extracorporeal membrane oxygenation (ECMO).

**Case summary:**

An 18-year-old Caucasian male was admitted due to fulminant myocarditis and signs of cardiogenic shock. He had to be put on venoarterial ECMO only hours after admission. Due to the occurrence of disseminated intravascular coagulation and heparin-induced thrombocytopenia, haemodynamic support was discontinued on Day 8. On Day 11 of his stay, the patient started to exhibit signs of severe infection and a single 1500 mg dose of azithromycin was prescribed. Immediately after starting the infusion, the patient developed profound hypotension and signs of cardiogenic shock. Consecutively, venoarterial ECMO had to be re-established, and the azithromycin infusion was stopped in the process. It took the restart of the compound to recognize the connection between the administration of the therapy and the occurrence of cardiogenic shock. After discontinuing azithromycin, no further sudden hypotensive episodes were recorded, and the patient received left ventricular assist device implantation as a bridge to recovery or transplant.

**Discussion:**

Rapid-onset hypotension appears to be a very rare but important adverse drug reaction associated with intravenous administration of azithromycin. Factors such as preceding infection and reduced biventricular function may facilitate the described occurrence.

**Learning points**

- Rapid-onset hypotension appears to be a very rare but important adverse drug reaction associated with intravenous administration of azithromycin.
- Factors such as preceding infection and reduced biventricular function may facilitate the occurrence of hypotension in patients receiving azithromycin.

## Introduction

Azithromycin is commonly used to treat out-of-hospital pneumonia and has been utilized in cases with mycoplasm myocarditis.^1^ A well-known adverse effect of azithromycin is prolongation of the QT interval, potentially leading to life-threatening Torsade de Pointes arrhythmias, as outlined in the label and also by large health systems, such as the UK National Health Service (NHS). However, awareness is limited with regard to severe hypotension and shock as potential adverse effects.^2^ In the past, administration of azithromycin has been linked to severe hypotension in some case reports.^1,3^ We herein report a further case of profound shock requiring excessive vasopressors and extracorporeal membrane oxygenation (ECMO) following intravenous treatment with azithromycin in a young patient with fulminant myocarditis.

## Summary figure

Rapid-onset hypotension is a rare but significant side effect that can occur after intravenous azithromycin administration. Ongoing infection and reduced biventricular function may act as additional risk factors that increase the likelihood of the reaction.

**Figure:**
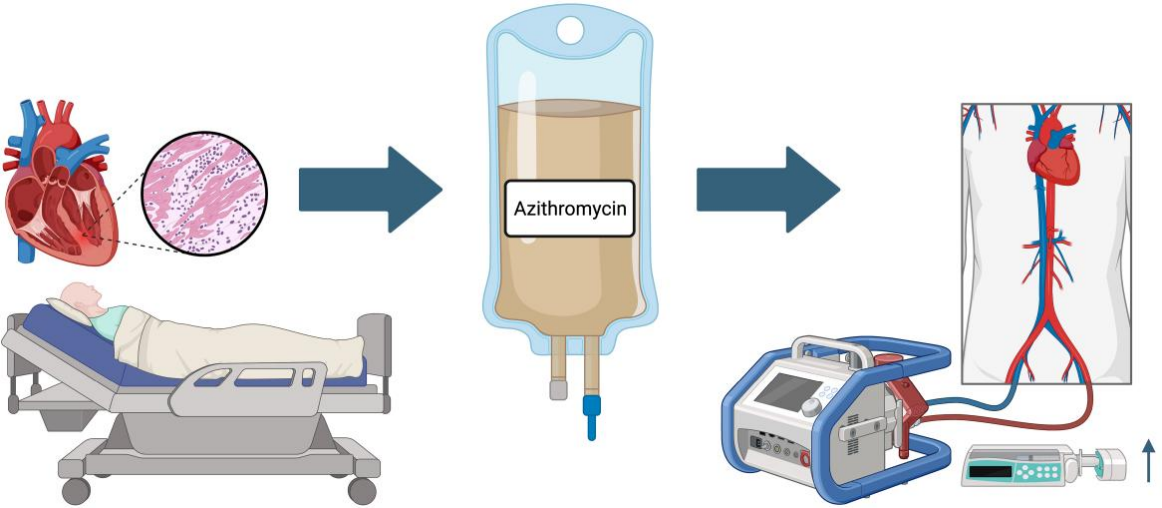


## Case presentation

An 18-year-old Caucasian male was transferred to our tertiary care centre due to suspected fulminant myocarditis. He exhibited profound cardiogenic shock associated with acute kidney failure (creatinine on admission 2.17 mg/dL, urine output 230 mL/24 h) and ischaemic hepatitis (peak aspartate aminotransferase 2267 U/L, bilirubin 1.8 mg/dL). Despite receiving levosimendan, dobutamine, and norepinephrine, the patient further deteriorated, with serum lactate levels increasing steadily to 6.8 mmol/L. Consequently, the patient was put on venoarterial ECMO only hours after admission. In the 3 weeks leading up to the event, the patient had experienced fever, fatigue, and mild respiratory symptoms, and his previous medical history was otherwise unremarkable. Upon admission, his right and left ventricular function was found to be severely depressed, and there were two left-sided intraventricular thrombi measuring 31 × 14 and 24 × 10 mm, respectively. In the days ensuing, elevated D-dimer levels were found peaking at 100.4 µg/mL along with a drop in fibrinogen levels (with a minimum of 94 mg/dL), a drop in platelet count to 25 G/L, and the haemolysis index dramatically increased (corresponding to a free haemoglobin level of 63.3 mg/dL). Multiple thrombi were visible in the ECMO circuit, primarily located in the venous ECMO cannula. While the laboratory results were suggestive of severe disseminated intravascular coagulation (DIC), antibodies against platelet-factor-4–heparin complexes turned out to be highly positive, indicating an additional occurrence of heparin-induced thrombocytopenia (HIT). In the presence of multiple thrombi, ongoing DIC, haemolysis, and slightly improved haemodynamics, an urgent decision was required and venoarterial ECMO treatment was discontinued on Day 8. This decision was supported by the absence of vasopressors, recovering hypoxic hepatitis, and normal urine output. The anticoagulation regimen was switched to argatroban. Subsequently, the patient was stable on dobutamine (1.5 µg/kg/min) and norepinephrine (0.120 µg/kg/min) for the ensuing 3 days.

On Day 12 of his intensive care unit (ICU) stay, the patient presented septic, with mental confusion, tachypnoea, and a sharp rise in leukocytes to 28.3 G/L and CRP 24.3 mg/dL. Up to this point, multiple blood cultures turned out negative (one was found positive for *Staphylococcus epidermidis* with a very low bacterial count and considered the result of contamination). The patient already received a combination of daptomycin and piperacillin/tazobactam for 7 days while under ECMO treatment. This treatment was continued, and the rise in inflammatory markers occurred despite an adequate dosage of both antimicrobial agents. In the absence of a biopsy result, the possibility of mycoplasma-induced myocarditis was considered. Therefore, a single 1500 mg dose of azithromycin was prescribed by the infectiologist. The patient showed some improvement through the use of crystalloids and vasopressors with a minimum lactate of 4.5 mmol/L. However, immediately after starting the azithromycin infusion, the patient developed profound hypotension and signs of cardiogenic shock. Serum lactate levels rose again, and catecholamine doses had to be increased drastically. Azithromycin and intravenous fluid therapy were stopped due to haemodynamic deterioration and pulmonary oedema. The patient did not show any signs of bronchospasm, skin rash, or angioedema. His condition worsened continuously, and following a rise of lactate to 9.3 mmol/L, the decision was made for intubation and re-implantation of venoarterial ECMO. With re-established extracorporeal haemodynamic support, stabilization was achieved, and lactate levels fell to 3.2 mmol/L with continuous reduction of vasopressors. Because azithromycin had been stopped earlier, the patient did not receive the full dose, and the compound was re-administered later the same day. After starting the infusion for the second time, pulsatility on invasive blood pressure monitoring (i.e. intrinsic cardiac activity) was promptly lost. A second arterial line confirmed a pulseless blood pressure curve. Continuous electrocardiogram (ECG) monitoring revealed no arrhythmic events as a potential cause of haemodynamic compromise. Again, the patient developed fulminant cardiogenic shock characterized by rapidly increasing lactate levels and significantly reduced mean arterial pressure despite a high ECMO flow of 5.4 L per minute. The patient required peak doses of 0.9 µg/kg/min norepinephrine, 4 IE/h vasopressin, 8.4 mg/h hydrocortisone, and 8.0 µg/kg/min dobutamine. In this situation, the infusion of azithromycin was recognized as the triggering factor and stopped subsequently. Over the next 12 h, catecholamines could be reduced to a minimum, and lactate levels were found below 2.0 mmol/L. After discontinuing azithromycin, there were no further sudden hypotensive episodes.

A myocardial biopsy of the left ventricle revealed moderate lymphocytic infiltration without evidence of giant cell myocarditis, sarcoidosis, or any other specific origin. Therefore, viral myocarditis was assumed as the most probable underlying cause. In view of his young age and the critical situation, the patient was primarily listed for urgent cardiac transplant. However, because of the lack of available donor organs within a reasonable time frame (the patient’s blood type being O), a left ventricular assist device (LVAD) had to be implanted. Finally, on Day 49 of his ICU stay, the patient was transferred to an intermediate care unit for further rehabilitation. From there, the patient was discharged with the LVAD system as bridge to recovery or transplant.

## Discussion

Herein, we present a case of assumed azithromycin-induced severe cardiogenic shock in a young patient with fulminant viral myocarditis. Few case reports associating intravenous administration of azithromycin with the occurrence of severe hypotensive episodes are available. Two cases reported children suffering from severe cardiogenic shock after receiving the compound as a treatment for mycoplasma myocarditis. In both instances, symptoms developed soon after the first intravenous application, and the patients died at the ICU or required implantation of ECMO. Before receiving azithromycin therapy, both patients had already exhibited significantly depressed biventricular function, as in our patient.^1^ Additionally, there is one report of an adult with a similar presentation following intravenous administration. A 64-year-old male Caucasian exhibited multiple episodes of severe hypotension closely linked to periodic administration of azithromycin.^3^ In this report, we present another case of a young patient with myocarditis experiencing severe cardiogenic shock twice after receiving azithromycin. The reported episodes appear much more likely to be an adverse drug reaction than being caused by drug-induced anaphylaxis. Our patient exhibited no allergic symptoms, such as angioedema or flush. There is evidence of acute hypotension occurring during co-prescription of calcium channel blockers and macrolide antibiotics. Yet, these reports do not specifically describe the use of azithromycin.^4,5^ Additionally, our patient did not receive calcium channel blockers. However, further drug interactions cannot be ruled out. In particular, effects on myocardial calcium homeostasis and intrinsic cardiac activity might cause the observed occurrence of shock. Especially, this may come into effect in the presence of pre-existing biventricular compromise. Further studies are required to uncover potential molecular mechanisms involved in this context. Furthermore, a meta-analysis on macrolides revealed a higher incidence of arrhythmias.^6^ In our case, continuous ECG monitoring did not reveal abnormalities during the hypotensive episodes, ruling out arrhythmic events. In conclusion, rapid-onset hypotension appears to be a very rare but important adverse drug reaction associated with intravenous administration of azithromycin. Factors such as preceding infection and reduced cardiac function may facilitate the described occurrence, and particular caution should be taken when using azithromycin in patients with these pre-existing conditions.

## Data Availability

The data underlying this article will be shared on reasonable request to the corresponding author.

## Lead author biography

Max Lenz works at the Medical University of Vienna (Department of Cardiology). His areas of expertise are intensive care medicine and translational research, with a focus on inflammation in different disease settings. His current scientific work encompasses topics like cardiogenic shock, fulminant myocarditis, atherosclerosis, and the role of innate immune cells in these conditions.
